# Upregulation of SPINK2 in acute myeloid leukemia

**DOI:** 10.1515/almed-2022-0047

**Published:** 2023-02-20

**Authors:** Sümbül Gezer, Zeliha Emrence, Tuğrul Elverdi, Muhlis Cem Ar, Burcu Salman Yaylaz, Ferda Paçal, Ayşegül Ünüvar, Melda Sarıman, Ahmet Emre Eşkazan, Serap Karaman, Ayşe Salihoğlu, Zeynep Karakaş, Neslihan Abacı, Sema Sırma-Ekmekci

**Affiliations:** Department of Genetics, Istanbul University, Aziz Sancar Institute of Experimental Medicine, Istanbul, Türkiye; Istanbul University, Institute of Graduate Studies in Health Sciences, Istanbul, Türkiye; Department of Internal Medicine, Cerrahpasa Faculty of Medicine, Division of Hematology, Istanbul University-Cerrahpasa, Istanbul, Türkiye; Division of Pediatric Hematology and Oncology, Istanbul University, Istanbul Faculty of Medicine, Istanbul, Türkiye

**Keywords:** acute myeloid leukemia, AML, HUSI-II, serin protease inhibitors, SPINK2

## Abstract

**Objectives:**

Acute myeloid leukemia (AML) is a highly heterogeneous disease. Although patients can be classified into risk groups based on their genetic changes, the prognosis of disease within these categories varies widely. This situation raises the need to search for new molecular markers related to AML. Serine peptidase inhibitor Kazal type 2 (*SPINK2*) has recently been reported to be upregulated in AML and associated with poor outcomes by meta-analysis and in a limited number of AML patients.

**Methods:**

We analyzed *SPINK2* mRNA expression in 62 patients (45 adult and 17 pediatric) with AML and 11 cell lines using quantitative Real-Time PCR (qRT-PCR). SPINK2 protein level was determined using ELISA in cell lines.

**Results:**

We found that the expression of *SPINK2* mRNA and protein levels in AML cell lines (HL60 and NB4) have increased compared to other cell lines (K562, Jurkat and NALM6, MCF7, HeLa, HUVEC, hFOB, 293T, U87). *SPINK2* mRNA expression was upregulated in patients with AML compared to controls (p=0.004) and significantly lower in t(8;21)-positive patients compared to negative patients (p=0.0006).

**Conclusions:**

Our results suggest that *SPINK2* serves an important role in AML development. Further studies are needed to evaluate SPINK2 expression in AML patients with t(8.21) and investigate to clarify its prognostic value in various subgroups of AML.

## Introduction

Acute myeloid leukemia (AML), a rapidly progressing malignant disease, is a clonal hematopoietic stem cell disorder characterized by an increase in the number of myeloid cells in the bone marrow and a block in their maturation. Its incidence increases with age [1, 2]. AML is genetically, biologically and clinically heterogeneous. Recurrent genetic alterations observed in AML are chromosomal alterations such as the loss of part or all of the chr 5 and chr 7, *t*(15;17)(q22;q12), t(8;21)(q22;q22.1), inv(16)(p13.1q22)/t(16;16)(p13.1;q22), inv(3)(q21.3q26.2)/t(3;3)(q21.3;q26.2), t(6;9)(p23;q34.1), t(9;11)(p21.3;q23.3) and t(1;22)(p13.3;q13.3) [3]. Besides these chromosomal alterations, the most common mutations are in the *FLT3, NPM1, DNMT3A, KIT, CEBPA, RUNX1, IDH1/2 TET2 ve MLL, ASLX1, BCOR, BCORL1, TP53, GATA2, DDX41, KMT2A, SRP72 and STAG2* genes [4, 5].

*SPINK2* (*HUSI-II, ISK2*), a member of the Kazal-type serine protease inhibitor family, functions as an acrosine-trypsin inhibitor. *SPINK2* expression is associated with fertility [6, 7]. Tazarotene-induced gene 1 (TIG1) encodes a protein that is a retinoid-regulated tumor suppressor. SPINK2 affected TIG1-regulated urokinase-type plasminogen activator (uPA) activity, which regulates invasion, metastasis and epithelial-mesenchymal transition (EMT) [8]. *SPINK2* expression increases significantly in retinal ganglion cells after optic nerve damage [9]. *SPINK2* mutation increases sensitivity to staurosporine-induced apoptotic stimuli [10]. The absence of SPINK2 induces a microautophagy-like pathway in germ cells [11].

*SPINK2* is one of the differentially expressed genes in primitive hematopoietic stem cells obtained from the umbilical cord blood [12]. *SPINK2* is significantly upregulated in the CD33+ bone marrow (BM) blast cells of a patient with AML compared to normal BM CD33+ cells in a case report [13]. It has been shown that *SPINK2* is upregulated gene in AML in data from the Gene Expression Omnibus (GEO) database [14]. The results of this study showed that elevated *SPINK2* expression was associated with poor outcomes [14]. This data need to be confirmed by further studies. This study aimed to determine the expression level of *SPINK2* and to evaluate its prognostic value in AML.

## Materials and methods

### Patient samples

In this retrospective study, 62 patients who were diagnosed at Istanbul University, Istanbul Medical Faculty, Pediatric Hematology Oncology and Department and Istanbul University-Cerrahpasa, Cerrahpasa Medical Faculty, Department of Internal Medicine, Division of Hematology were included. Blood or bone marrow samples were obtained for routine analysis with the informed consent from 17 pediatric (under 18 years) and 45 adult (18–80 years) patients with AML at diagnosis between 2013 and 2016. *SPINK2* expression analysis was performed using leftover RNA samples of patients from routine fusion gene analysis. The control group consisted of three pediatric (under 18 years) and five adults (26–67 years old), who were bone marrow transplantation donors. Apart from healthy control, nine individuals without leukemia who underwent bone marrow biopsy due to the pre-diagnosis of leukemia were also used as another group of controls. The approval of Istanbul University Istanbul Medical Faculty Clinical Research Ethics Committee (reference number: 537, 24/05/2017) was obtained for this study. This study was conducted in accordance with the 1964 Declaration of Helsinki and its subsequent amendments. The demographic characteristics of the patients are shown in Table 1.

**Table 1:** Clinical and biological characteristics of the patients with AML.

	Pediatric AML	Adult AML
Median age, years	9.5 (range: 1.1–80)	50.44 (range: 18–80)
Sex, male/female	29/33	22/23
Median WBC, cell/µL	8,600 (range: 2,100–1,80,000)	311,700 (range: 1,000–1,80,000)

FAB classification, n		

M0	1	1
M1	3	3
M2	7	5
M3	10	4
M4	12	7
M5	3	3
M6	0	0
M7	0	0
NA	26	22

Genetic alterations, n		

t(15;17)-positive	12	6
t(8;21)-positive	11	8
inv(16) or t(16;16)-positive	6	4
Negative	33	27

AML, acute myeloid leukemia; WBC, white blood cell; FAB, French American British; M0–M7, patient’s FAB, groups; NA, not available.

### Cell lines

HL60 (acute promyelocytic leukemia), NB4 (human promyelocytic leukemia), K562 (chronic myeloid leukemia), Jurkat (acute T-cell leukemia) and NALM6 (acute lymphoblastic leukemia) cell lines were cultured in RPMI medium containing 10% FBS, 2 mM L-glutamine, 100 units/mL penicillin and 100 μg/mL streptomycin. MCF7 (epithelial cell obtained from mammary gland metastatic area), HeLa (cervical epithelial cell with adenocarcinoma), HUVEC (endothelial cell derived from the human umbilical cord), hFOB (human osteoblasts), 293T (human embryonic kidney epithelial cell), U87 (human brain epithelial cell line) were cultured in the DMEM medium, including 10% FBS, 2 mM L-glutamine, 100 units/mL penicillin and 100 μg/mL streptomycin. They were reproduced in a 5% CO_2_ incubator at 37 °C.

### Determination of *SPINK2* mRNA expression in patients with AML and cell lines using qRT-PCR

RNA samples were extracted from cells using PureLink, RNA Mini Kit (Invitrogen, Carlsbad, CA, USA) following the protocol of the kit. Transcriptor High Fidelity cDNA Synthesis Kit (Roche Diagnostic, Mannheim, Germany) was used for RT-PCR according to the manufacturer’s instructions. cDNA was synthesized from 500 ng total RNA in 20 µL reaction volume.

A real-time quantitative PCR device (LightCycler, Roche Diagnostic, Mannheim, Germany) was used for quantitation. TATA binding protein (TBP) housekeeping gene was used as a control gene. Primer sequences and PCR product sizes are given in Table 2. SYBR Green master kit (Roche Diagnostic, Mannheim, Germany) and 1 µL cDNA sample were used for the 20 µL PCR reaction. The PCR conditions were as follows 60 s pre-denaturation at 95 °C, 10 s at 95 °C, 20 s at 68 °C and 10 s at 72 °C (45 cycles). After amplification, the program was applied for melting curve analysis for 0 s at 95 °C, 10 s at 65 °C and 0 s at 95 °C. The differences in *SPINK2* expression in patients were determined using ∆∆Ct method according to the healthy control sample [15]. mRNA expression levels were indicated as arbitrary units (AU).

**Table 2:** Primer sequences and PCR product sizes.

Gene name	Primers	PCR product size
*SPINK2*	Forward: ACCAGGATGTCCCAGACACT	179 bp
	Reverse: GCCAGTGAAGGTGGTCTCTC	
*TBP*	Forward: ACTTGACCTAAAGACCATTGCAC	120 bp
	Reverse: CTTGAAGTCCAAGAACTTAGCTGG	

### Determination of SPINK2 protein level using ELISA

The cultured cells were seeded with two million cells in 4 mL medium containing 0.2% FBS suitable for the cell type. After 48 h, cells were lysed in 200 µL of extraction solution (100 mM Tris, pH 7.4, 150 mM NaCl, 1 mM EGTA, 1 mM EDTA, 1% Triton X-100, 0.5% Sodium deoxycholate). Total protein concentration in cells was determined using the bicinchoninic acid assay (Pierce BCA Protein Assay kit, Thermo Fisher Scientific, Waltham, MA, USA). The Sandwich ELISA was performed according to the human serine protease inhibitor Kazal-type 2 (SPINK2) ELISA Kit (MyBioSource, San Diego, CA, USA) protocol. Samples were measured using the ELISA plate reader at 450 nm. Intracellular SPINK2 protein levels were normalized to total protein levels. All samples, standards and controls were run in triplicate. The mean value of triplicate samples was calculated.

### Detection of fusion genes by RT-PCR analysis

AML1-ETO associated with t(8;21), CBFB-MYH11 with inv(16) and PML-RARA with t(15;17) fusion gene transcripts were detected by reverse transcriptase polymerase chain reaction (RT-PCR) [16].

### Statistical analysis

The data were analyzed using the SPSS Statistics Program Version 20.0 (IBM SPSS Statistics for Windows, Version 20.0. Armonk, NY: IBM Corp.) and GraphPad Prism 7.0. Kolmogorov-Smirnov and Shapiro-Wilk tests was used to determine normality. Independent sample T-test for parametric data and Kruskal-Wallis and Mann-Whitney U tests were used for non-parametric data. To investigate the correlation, Spearman’s tests were used. Receiver operating characteristic curve (ROC) and the area under curve (AUC) were conducted to assess the value of *SPINK2* expression for discriminating patients with AML from controls. Overall survival (OS) was defined as time from disease diagnosis to death from any cause or censoring for patients alive at their last known date of contact. The Kaplan-Meier analysis and log rank test were used to determine patients’ survival rates. p<0.05 was considered statistically significant for all statistical analyses.

## Results

### *SPINK2* expression in cell lines

qRT-PCR was used to determine the level of *SPINK2* mRNA in 11 cell lines (Supplementary Figure 1). We were found that the expression of *SPINK2* mRNA was higher in AML cell lines (median: 10.5 AU, range: 8.72–12,32 AU) than non-AML cell lines (median: 0.9 AU, range: 0.41–3.41 AU) using Mann-Whitney U test (p=0.036). *SPINK2* mRNA expression in leukemia cell lines (median: 3.4 AU, range: 2.39–12.32 AU) also significantly increased compared to non-leukemia cell lines (median: 0.7 AU, range: 0.41–1.32 AU) (p=0.006).

SPINK2 protein levels found in the cell lines and proteins secreted into the medium were determined by ELISA method. The intracellular and extracellular SPINK2 protein levels are given in Supplementary Figure 1. The intracellular and extracellular SPINK2 expression in AML cell lines (median: 0.074 ng/mg, range: 0.95–1.04 ng/mg and median: 3.85 ng/mg, range: 0.05–0.30 ng/mg, respectively) also significantly increased compared to non-AML cell lines (median: 0.056 ng/mL, range:0.05–0.079 ng/mL and median: 0.004 ng/mL, range: 0–0.02 ng/mL, respectively) (p=0.036 and p=0.023, respectively).

*SPINK2* mRNA was correlated with intracellular and extracellular protein (p=0.0002, R=0.935 and p=0.046, R=0.610 respectively). There was also a statistically significant correlation between the intracellular and secreted protein levels (p=0.002, R=0.817).

### *SPINK2* expression and its prognostic significance in patients

**Figure 1:**
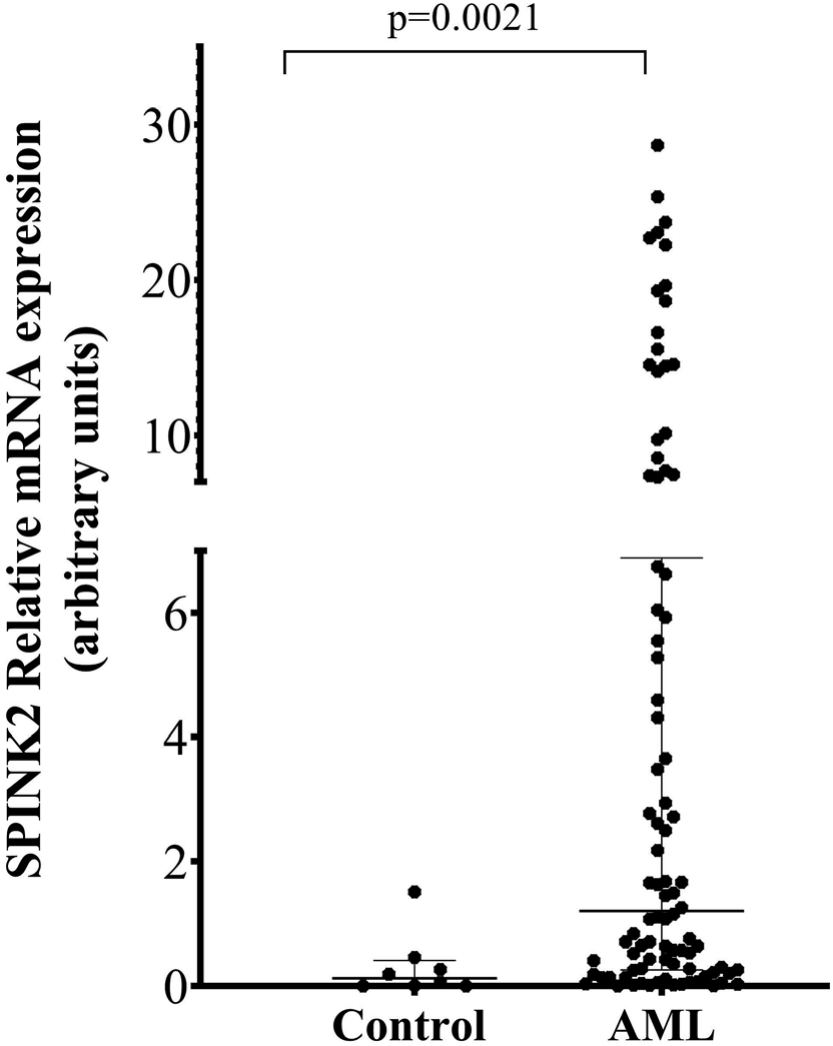
The median expression level of SPINK2 mRNA in healthy controls and patients with AML. SPINK2 mRNA levels were determined using cDNA derived from 25 ng total RNA. The relative mRNA expression levels were calculated using the comparative CT method. The long horizontal lines above the dots represent the median. The short horizontal lines above the dots represent the median interquartile range. AML, acute myeloid leukemia.

*SPINK2* mRNA levels in patients with AML (median: 1.62 AU, range: 0.0032–28.67 AU) were significantly higher than in controls (median: 0.058 AU, range: 0.00017–1.51 AU), which showed that *SPINK2* expression was significantly up-regulated in AML (p=0.004) (Figure 1). We also used nine samples from individuals without leukemia who underwent bone marrow biopsy due to the pre-diagnosis of leukemia as another group of control apart from healthy control samples. The median value of *SPINK2* mRNA expression was 0.28 AU (range: 0.035–1.15 AU). We observed that SPINK2 was significantly increased in AML, even compared with nine samples without leukemia (p=0.022).

**Figure 2:**
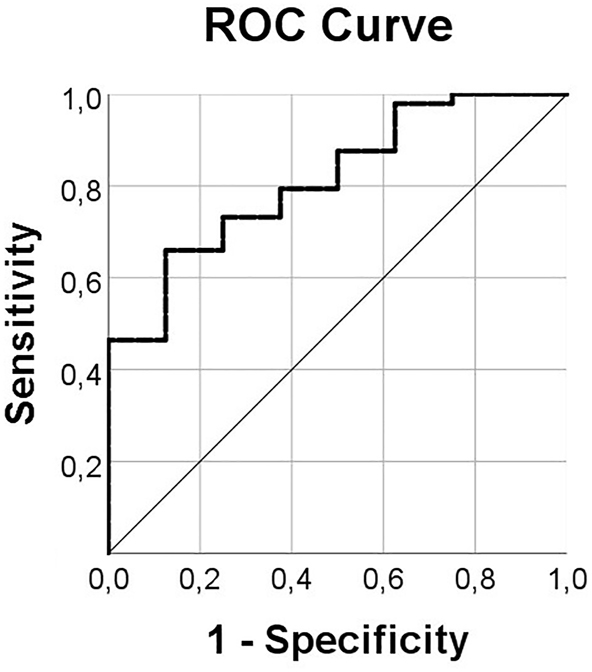
ROC curve of SPINK2 mRNA expression in AML. AML, acute myeloid leukemia.

Additionally, ROC curves were generated to evaluate the diagnostic value of *SPINK2* expression in AML. ROC analysis revealed that *SPINK2* is a well diagnostic marker with the AUC value of 0.82 [95% confidence interval (CI): 0.685–0.946] (p=0.004). The result is shown in Figure 2.

The comparisons of *SPINK2* expression according to the clinical and laboratory characteristics of patients with AML are given in Table 3. There was no significant relationship between *SPINK2* expression and age, gender, white blood cell count and French American British (FAB) groups in patients (Table 3).

**Table 3:** Comparison of *SPINK2* expression with respect to the clinical and laboratory features of pediatric patients with AML.

	Pediatric AML			Adult AML		
		n	*SPINK2*		n	*SPINK2*
			p-Value			p-Value
Age, year	10>	10	0.699	60>	31	**0.044**
	10<	7		60<	14	
WBC, cell/µL	10,000>	7	0.527	50,000>	19	0.821
	10,000<	4		50,000<	7	
	NA	6		NA	19	
Gender	Male	7	0.669	Male	22	0.946
	Female	10		Female	23	
FAB	M0	0	0.562	M0	1	0.105
	M1	0		M1	3	
	M2	2		M2	5	
	M3	6		M3	4	
	M4	5		M4	7	
	M5	0		M5	3	
	M6	0		M6	0	
	M7	0		M7	0	
	NA	4		NA	22	
Genetic alterations	t(15;17)	6	0.776	t(15;17)	6	0.317
	t(8;21)	3	0.057	t(8;21)	8	**0.00005**
	inv(16)	2	0.059	inv(16)	4	0.322
	Negative	6		Negative	27	

p-Values marked with bold indicate statistically significant differences between the groups. AML, acute myeloid leukemia; WBC, white blood cell; FAB, French American British; M0–M7, patient’s FAB, groups; NA, not avaiable.

*SPINK2* expression was analysed in AML patients with t(15;17), t(8;21), inv(16) mutations using the Mann Whitney U test (Table 3). A statistically significant difference was found between t(8;21)-positive and negative patients. *SPINK2* expression was significantly lower in t(8;21)-positive than in negative patients with AML (p=0.00006).

**Figure 3:**
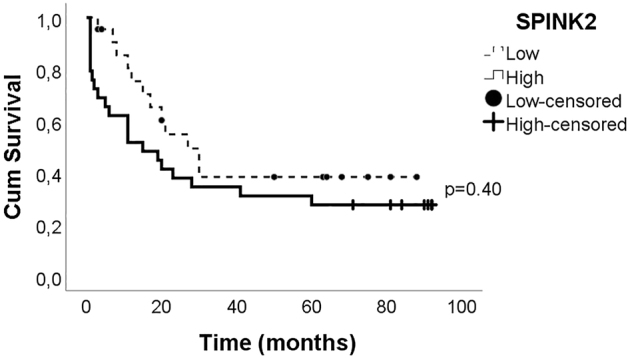
Impact of the SPINK2 expression on the overall survival rate of patients with AML. Overall survival was evaluated by the Kaplan-Meier curve. The cut-off value was determined at the median expression level (1.62 AU) to define the groups. AML, acute myeloid leukemia.

We categorized the patients (n=50) into two groups (*SPINK2* ^high^ and *SPINK2* ^low^) based on the median expression level of *SPINK2* mRNA (1.62 AU) to evaluate the prognostic value of *SPINK2* expression. According to Kaplan-Meier analysis, the 7-year overall survival rates were 27.9 ± 8.4%, for SPINK2^high^ and 40.6 ± 11.6% for *SPINK2* ^low^ patients. There was no significant difference in OS between *SPINK2* ^high^ and *SPINK2* ^low^ patients (log-rank p= 0.40) (Figure 3). Median survival times were 15 (range: 1–92) months for *SPINK2* ^high^ and 20.5 (range: 3–89) months for *SPINK2* ^low^ patients.

## Discussion

Acute myeloid leukemia (AML) is a heterogeneous disease that results in abnormal hematopoiesis, block in differentiation, excessive proliferation of myeloblasts, and disruption of normal blood cell production [17]. Although the presence of various mutations is used as a prognostic factor for the classification of the patients, molecular subgroups of patients need further improvement. This situation raises the need to search for new molecular markers related to AML.

It has been reported that the *SPINK2* is overexpressed in hematopoietic progenitor stem cells [18]. He et al. identified *SPINK2* as one of the differentially expressed genes (DEG) when comparing CD34+ and CD133+ hematopoietic progenitor stem cells and CD34+ cells purified from umbilical cord blood [12]. Transcriptome analysis in CD33+BM blast cells purified from a patient with AML transformed from myelodysplastic syndrome showed that the expression of *SPINK2* was higher than control CD33+ BM cells [13].

In a recent study, to the identification of pivotal genes in AML, microarray data from Gene Expression Omnibus (GEO) analyzed via bioinformatics analysis [14]. They revealed that the expression level of *SPINK2* was the most altered among all upregulated genes. They validated this result using qRT-PCR in six controls and 12 patients with AML and simultaneously, in GEPIA (Gene Expression Profilling Interactive Analysis) and Oncomine data. They found that elevated *SPINK2* expression was associated with poor outcomes [14]. Barresi et al. found that *SPINK2* was overexpressed in NUP98+ patients from the data GEO database and 358 RNAseq AML samples from TARGET data. They also confirmed in pediatric patients with primary induction failure using qRT-PCR [19].

In our study, *w*e found that *SPINK2* mRNA was upregulated in pediatric, adult and total patients with AML, which was consistent with the results of the studies by Barresi et al. and Xue et al. [14, 19]. The ROC analysis showed that *SPINK2* expression might serve as a potential biomarker for distinguishing AML from controls.

*SPINK2* expression was evaluated in pediatric, adult and total AML patients with t(15;17), t(8;21) and inv(16) mutations. We found that *SPINK2* expression significantly decreased in t(8;21) positive patients compared to negative patients for both total and adult patients with AML. This is the first study to show a statistically significant association between t(8;21) and low *SPINK2* expression. t(8;21) is known to be associated with a favorable prognosis [3]. However, Xue et al. reported that high *SPINK2* expression is associated with poor prognosis [14]. We suggest that the high survival rate of *SPINK2* ^low^ patients might be related to t(8;21) positivity. The association between *SPINK2* expression and t(8;21) should be validated in large patient cohorts.

In this study, we were only analyzed *SPINK2* mRNA expression since we did not have protein samples from the patients. We could not detect the reflection of the *SPINK2* mRNA upregulation to the protein levels in patients with AML. Therefore, we analyzed SPINK2 expression at both mRNA and protein levels in 11 cell lines to investigate the correlation between *SPINK2* mRNA and protein levels. *SPINK2* mRNA levels were determined by qRT-PCR and the intracellular and secreted protein levels were determined by ELISA in cell lines. The expression of *SPINK2* mRNA in AML cell lines was significantly increased compared to other cell lines. It was also significantly increased in leukemia cell lines compared to non-leukemic cell lines. To understand different implications of this molecule in other hematopoietic neoplasms, *SPINK2* expression should also be investigated in acute lymphoblastic leukemia, myelodysplastic syndrome and myeloproliferative neoplasms.

The intracellular SPINK2 protein level was found to be significantly increased in AML cell lines compared to other cell lines. A statistically significant correlation was found between *SPINK2* mRNA levels and intracellular and secreted protein levels. There was also a statistically significant correlation between the levels of intracellular protein and secreted protein. The correlation between *SPINK2* mRNA and protein levels suggests that the elevated *SPINK2* mRNA expression results in increased protein activity.

Xue et al. analyzed the effect of SPINK2 on the prognosis of patients with AML using the GEPIA (106 patients) and UALCAN datasets (163 patients). They showed that *SPINK2* ^high^ patients had shorter survival times than *SPINK2* ^low^ patients [14]. In this study, there was no significant difference between *SPINK2* ^high^ and *SPINK2* ^low^ patients. Since we were able to perform survival analysis in a limited number of patients, no statistically significant difference could be found.

This study has some limitations. First, survival analysis was performed in a limited number of patients, since the outcomes of some patients were not available. Secondly, *SPINK2* expression was not analyzed in different molecular subtypes of AML because of mutation profiles of the patients were not available.

Our findings showed that *SPINK2* is upregulated in patients with AML and *SPINK2* expression is significantly low in t(8;21)-positive AML subgroup. It should be evaluated in large patient cohorts to clarify the prognostic significance of *SPINK2* expression. Further studies are required to investigate the potential role of *SPINK2* in the development of AML and *SPINK2* targeting therapies.

## Supplementary Material

Supplementary Material

Supplementary Material

Supplementary Material
