# Supplementary material for: Upregulation of SPINK2 in acute myeloid leukemia
Source: Adv Lab Med. 2023 Feb 20;4(1):92–7. doi: 10.1515/almed-2022-0047 (PMC10197194; doi:10.1515/almed-2022-0047)
Supplement: Supplementary file 1 — Supplementary Material [file j_almed-2022-0047_suppl_001.docx]

**Supplementary Figure 1.** SPINK2 in expression in human cell lines including AML cell lines. (A) SPINK2 relative mRNA expression. SPINK2 mRNA levels were determined using cDNA derived from 25ng total RNA. The relative mRNA expression levels were calculated using the comparative CT method. (B) SPINK2 protein levels. SPINK2 protein levels were measured using ELISA. Intracellular SPINK2 protein levels were normalized to total protein levels (ng/mg) and extracellular SPINK2 protein levels were normalized to volume (ng/mL).
