# Supplementary figures and images for: Upregulation of SPINK2 in acute myeloid leukemia
Source: Adv Lab Med. 2023 Feb 20;4(1):92–7. doi: 10.1515/almed-2022-0047 (PMC10197194; doi:10.1515/almed-2022-0047)

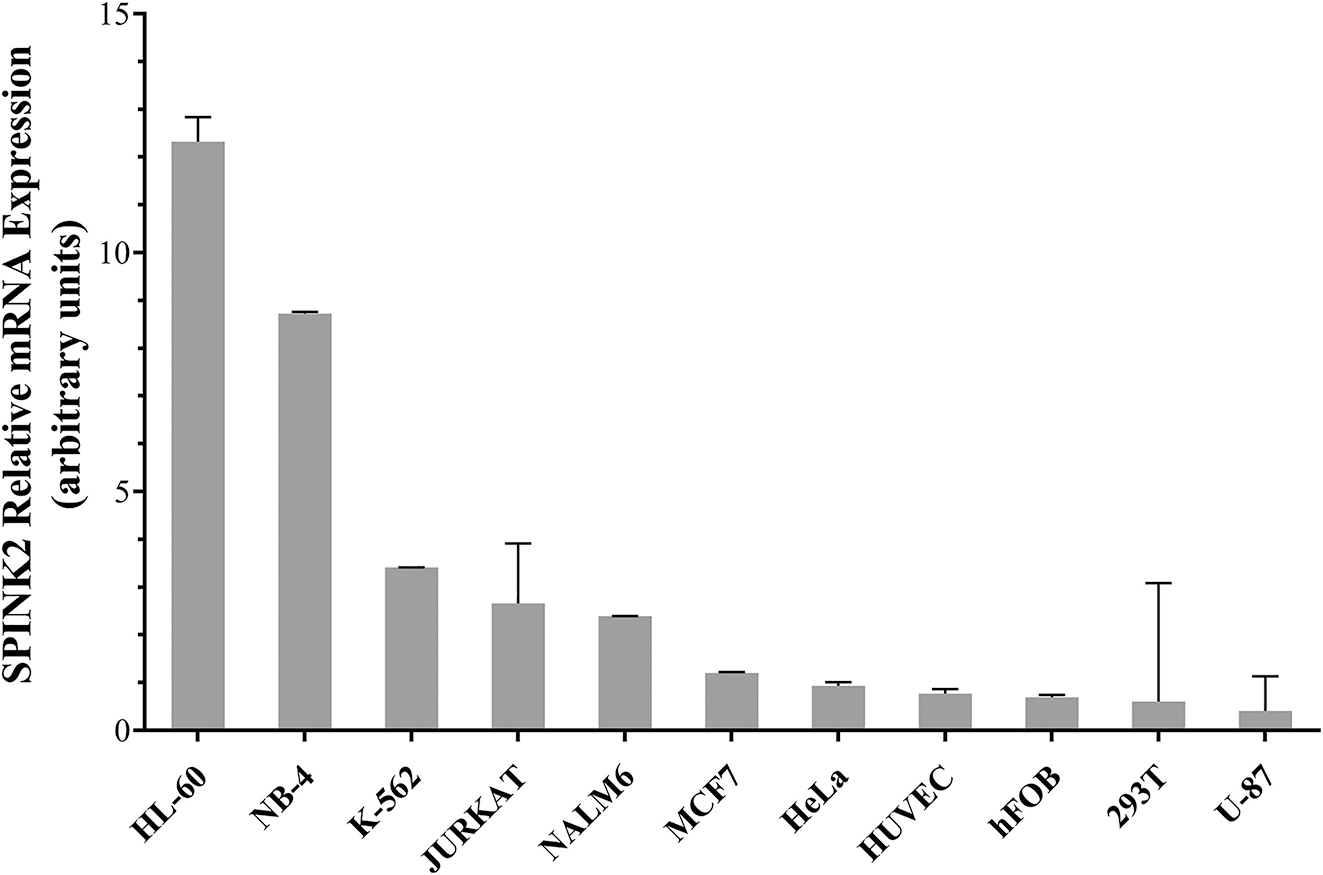

Supplement: Supplementary file 2 — Supplementary Material [file j_almed-2022-0047_suppl_002.jpg]

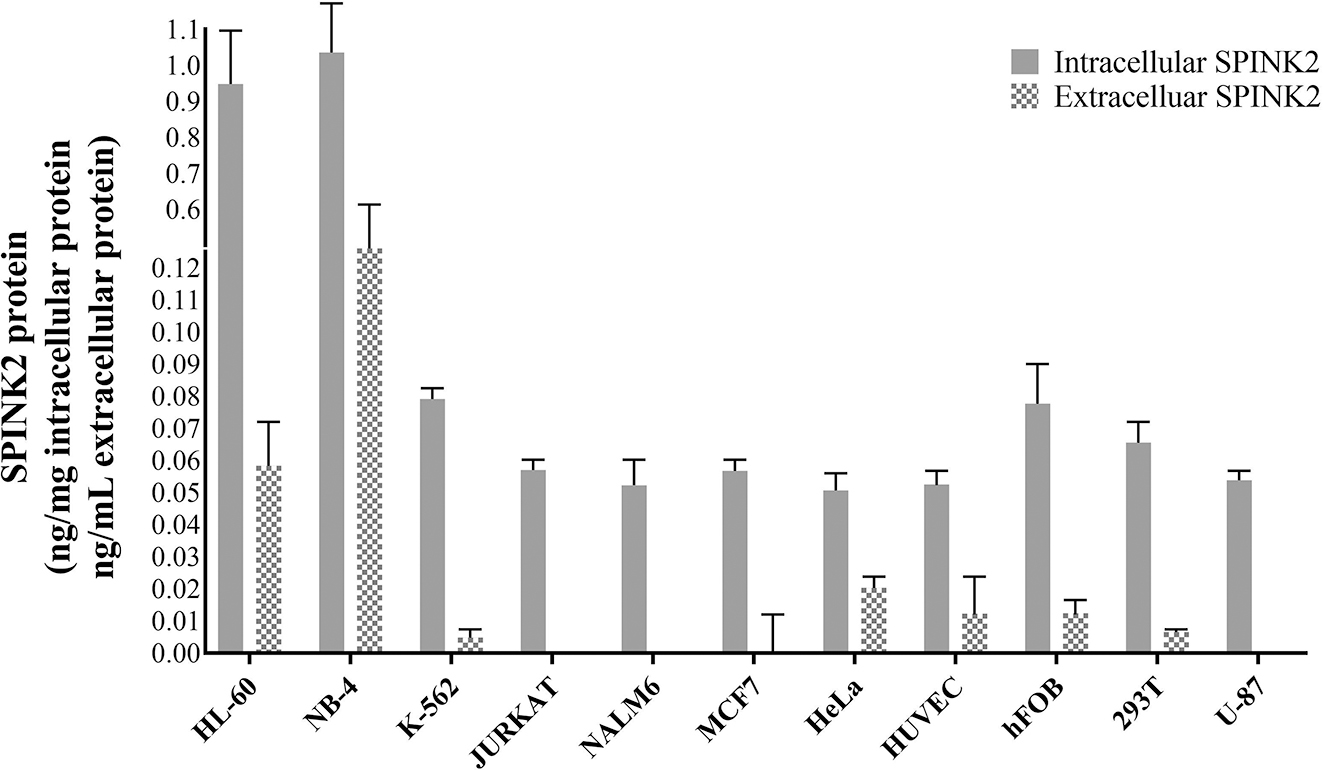

Supplement: Supplementary file 3 — Supplementary Material [file j_almed-2022-0047_suppl_003.jpg]
